# Immediate post partum macular subretinal bleeding in a highly myopic patient: a case report

**DOI:** 10.1186/s12886-021-01814-9

**Published:** 2021-01-21

**Authors:** Karen Bitton, J.-L. Bacquet, F. Amoroso, S. Mrejen, M. Paques, E. H. Souied

**Affiliations:** 1 Department of Ophthalmology, Quinze-Vingts National Hospital, Paris, France; 2 Department of Ophthalmology, Centre Hospitalier Intercommunal de Creteil, University Paris Est Creteil, Créteil, France

**Keywords:** Pathologic myopia, Lacquer cracks, Bruch’s membrane rupture, Pregnancy, Post partum, Subretinal bleeding

## Abstract

**Background:**

Pathologic myopia is a major cause of visual impairment and blindness.

**Case presentation:**

We report a case of an immediate post partum macular subretinal bleeding observed in a highly myopic patient. A 30-years-old woman presented two days after childbirth for sudden loss of vision in her right eye. Multimodal imaging showed macular hemorrhage masking a subtle yellowish linear lesion corresponding to lacker crack. Due to the lack of evidence for choroidal neovascularization, a simple clinical and imaging monitoring was recommended. Six weeks later, we noted an improvement in her best-corrected visual acuity and a decreased in size of the macular hemorrhage.

**Conclusions:**

This is the first case reporting a macular subretinal bleeding on macular lacquer cracks in a highly myopic patient in immediate post partum.

Valsalva maneuver associated with vaginal delivery could explain the occurrence of the hemorrhage associated with lacquer crack. However, natural history of pathological myopia could not be excluded.

## Background

High myopia (HM), defined as refractive error of at least − 6.00 diopters or an axial length ≥ 26 mm, is a major cause of visual impairment and blindness, especially in younger patients [1].

The prevalence of HM rapidly increased over the last 50 years, mostly in East Asia.

A recent review estimated that the HM prevalence would increase from 2.7 % of the world’s population in 2000 to 9.8 % by 2050 [1].

Pathologic myopia (PM) was recently reclassified by the” Meta-Analysis for Pathologic Myopia“ [2].

In this metanalysis, myopic maculopathy (MM) lesions were classified into five categories from “no myopic retinal lesions” (Category 0, C0), “tessellated fundus only” (C1), “diffuse chorioretinal atrophy” (C2), “patchy chorioretinal atrophy” (C3), and “macular atrophy” (C4). Three additional features were added to these categories as “plus signs”: lacquer cracks (LCs), myopic choroidal neovascularization (CNV), and Fuchs spot [2].

Based on this new classification, the term myopic maculopathy is used for C2 to C4 lesions, the presence of “plus” signs or a posterior staphyloma [2].

Lacquer cracks (LC) are considered to be the corresponding lesions of the healing process of the mechanical rupture of the complex including the retinal pigment epithelium (RPE), Bruch’s membrane (BM), and choriocapillaris (CC) [3].

The axial elongation of the globe is believed to be the main factor responsible of LC formation [3].

LCs are supposed to be a risk factor for choroidal neovascularization, a sight-threatening complication of advanced pathological myopia [3].

Pregnancy is known to lead to several ocular physiologic changes due to the activation of estrogen (E2) and progesterone receptors [4]. Decrease in corneal sensitivity and intraocular pressure, increase in both corneal thickness and curvature or ptosis are common findings during pregnancy [4]. The course of ocular disorders such as uveitis and diabetic macular edema can be modified by the hormonal impregnation [4].

In myopic pregnant women, a modest myopic shift is described, usually regressive after childbirth [4]. In the past, pathologic myopia was frequently considered as indication for cesarean section because of the risk of retinal detachment or retinal hemorrhage due to concomitant choroidal neovascularization in macular region during the increased efforts of labour [4]. However, following studies showed that there was no worsening of those conditions after vaginal delivery [4, 5].

To the best of our knowledge, this is the first case reporting an immediate post-partum macular subretinal bleeding in a highly myopic patient.

## Case presentation

A 30-year-old woman presented at Eye Emergency Department in Quinze-Vingts National Hospital two days after childbirth for sudden visual loss and metamorphopsia in her right eye (RE).

She had unremarkable medical history, except for uncomplicated pathologic myopia, and no family history ; her first pregnancy occurred with vaginal delivery without any complications.

This second vaginal delivery was faster with four maternal pushing efforts and no use of obstetrical forceps was required. The approximate duration of labor was about 4 hours.

At ophthalmic examination, her best-corrected visual acuity (BCVA) of the RE was 20/64 and 20/25 on the left eye (LE), with respective refractive error of -17.00 D and − 18.00 D.

On slit-lamp examination, the anterior segment was unremarkable on both eyes. Intraocular pressure was 17 mmHg in the RE and 18 mmHg in the LE.

Dilated fundus examination revealed, on both eyes, the presence of myopic maculopathy characterized by a tessellated fundus (C1) and posterior type II staphyloma ; macular LCs were noted on both eyes. The RE examination showed also the presence of a macular hemorrhage associated with a yellowish white line, corresponding to a macular LC.

**Fig. 1:**
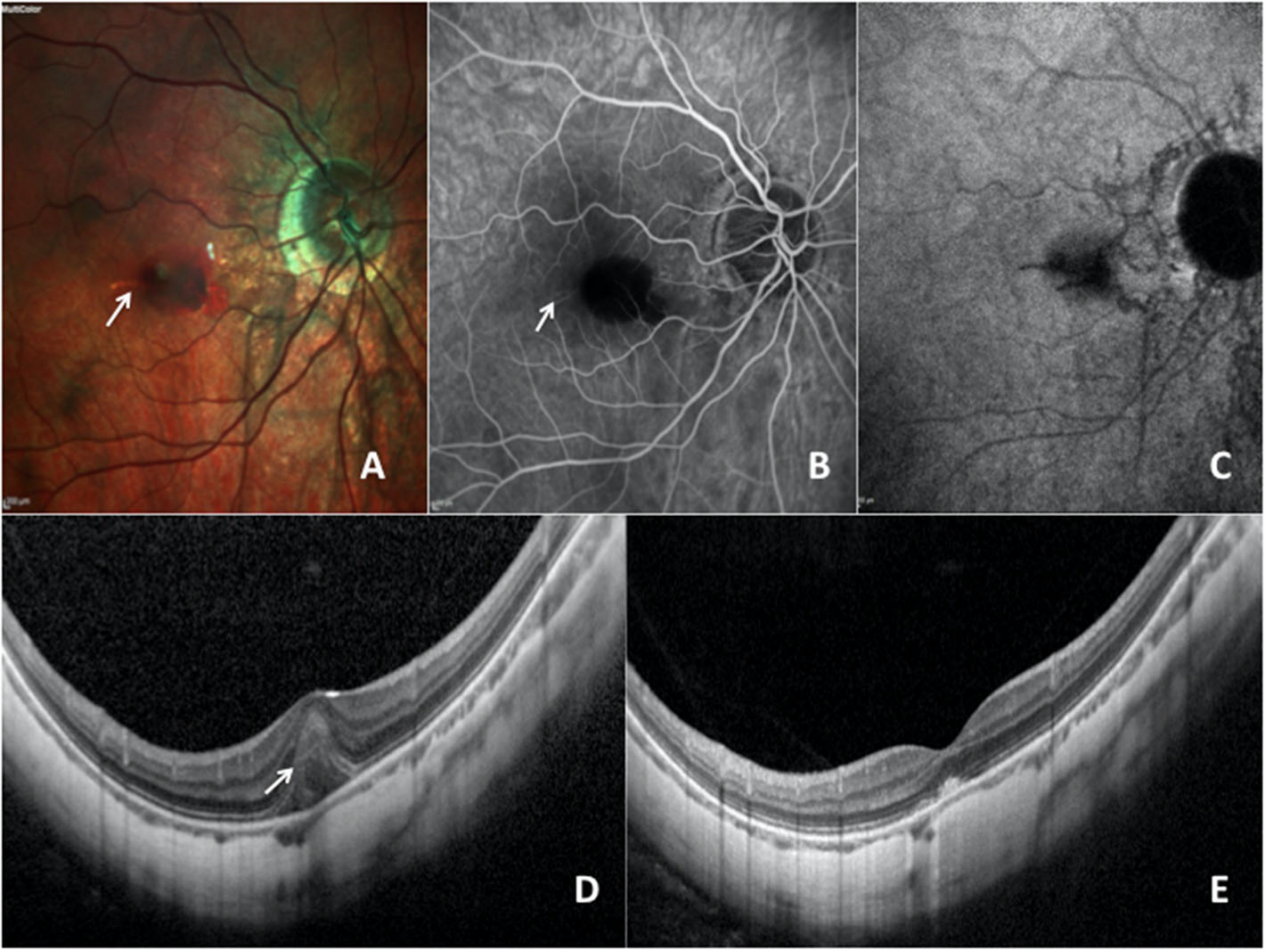
Multicolor fundus photograph, fluorescein angiography (FA), indocyanine green angiography (ICGA) and spectral domain optical coherence tomography (SD-OCT) of the right eye (RE) in post partum and 6 weeks later **a** MultiColor fundus photograph shows a macular hemorrhage masking a lacquer crack which appears as a yellowish horizontal line (*white arrow*). **b** FA. The macular hemorrhage blocks fluorescence, we can notice a discrete hyperfluorescent line (white arrow) **c** Late angiographic phase of ICGA showing hypofluorescence at the site of lacquer crack **d** SD-OCT. The vertical scanning line of macular lesion shows both an hyperreflective elevated lesion in the subretinal space (white arrow) and underlying irregularities of the RPE–BM–CC complex. **e** SD-OCT. Six weeks later, we can notice a reduction of the height of the subretinal hemorrhage with persistence of irregularities at the level of the RPE and the BM

The patient underwent multimodal imaging by HRA-Spectralis, Heidelberg Engineering (Fig. 1). Fluorescein angiography (FA) of the RE showed a macular hypofluorescence corresponding to the large macular hemorrhage. No dye leakage was noted. The LC appeared as a subtle horizontal linear hyperfluorescent lesion. Indocyanin green angiography (ICGA) showed a reticular hypofluorescent pattern at the site of lacquer cracks.

Spectral domain optical coherence tomography (SD-OCT) vertical B-scan revealed an elevated hyperreflective lesion in the outer retina due to subretinal hemorrhage with underlying irregularities of the RPE-BM-CC complex, suggestive of a lacquer crack.

**Fig. 2:**
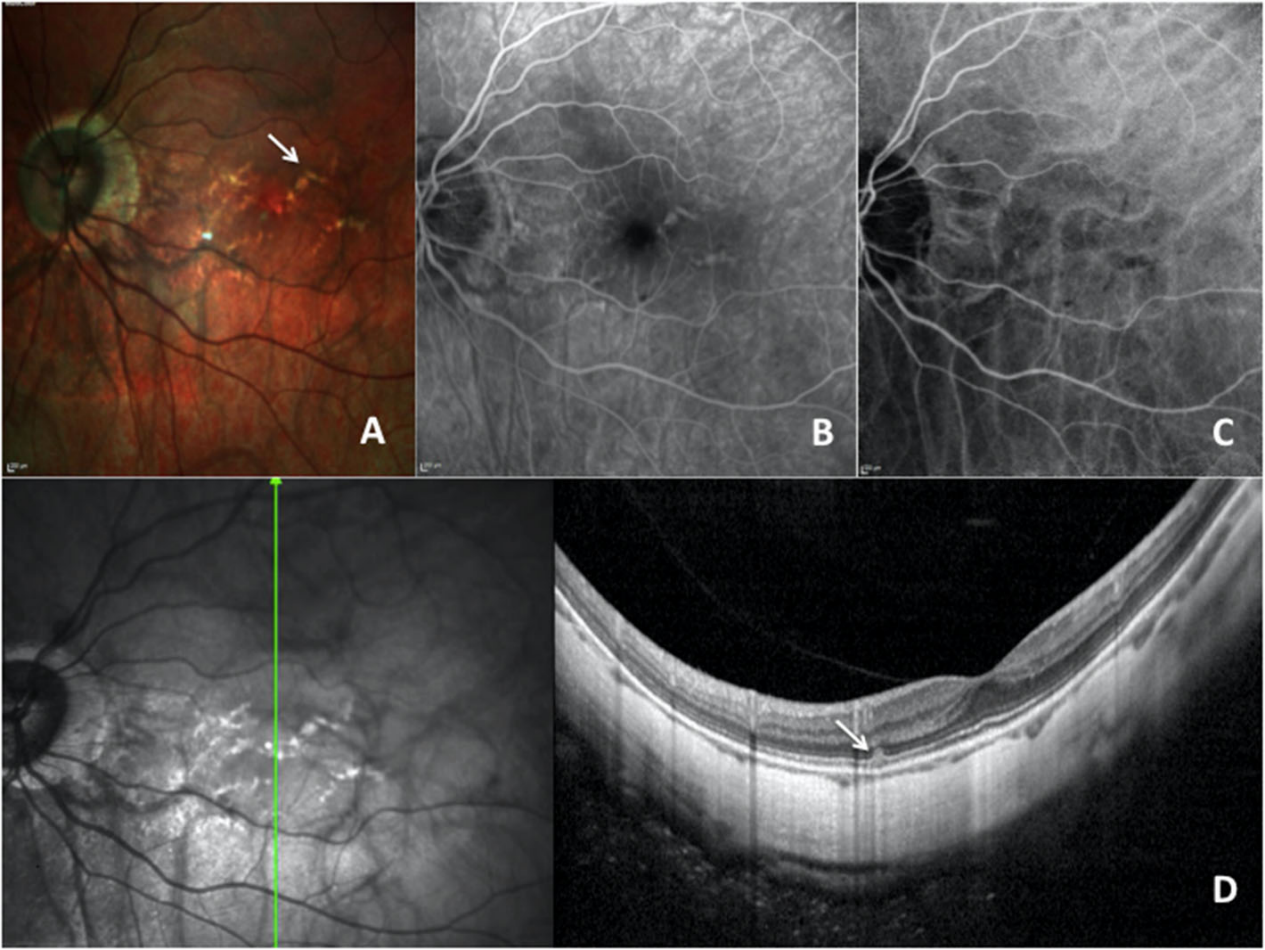
Fundus photograph, fluorescein angiography (FA), indocyanine green angiography (ICGA) and spectral domain optical coherence tomography (SD-OCT) of lacquer craks (LCs) of the left eye (LE). **a** Color photograph shows thick yellowish lines in a reticular pattern crisscrossing the posterior pole (white arrow). **b** fluorescein angiography shows hyperfluorescence at the site of the LCs. **c** Late angiographic phase of ICGA shows hypofluorescence at the site of the LCs. **d** SD-OCT reveals an elevated lesion at the level of the ellipsoid line and slightly irregular aspect of the RPE (white arrow)

Multimodal imaging on the LE (Fig. 2) was consistent with stellate macular LCs, tessellated fundus and posterior type II staphyloma. On FA, many LCs with a reticular hyperfluorescent pattern were observed. On the late phase of ICGA, LCs were hypofluorescent. SD-OCT showed discontinuities of the RPE-BM-CC complex with a depth increase in the OCT signal reflectivity.

Due to the absence of evidence for choroidal neovascularization (CNV), a simple clinical and imaging surveillance was recommended.

At six weeks follow-up, the patient reported an improvement in vision. Her BCVA improved from 20/64 to 20/32 in the RE.

Fundus examination showed a slight decrease in size of the macular hemorrhage.

SD-OCT vertical B-scan showed a decreased subretinal hemorrhage, with persistence of the irregularities of the RPE-BM-CC complex owing to the LC.

Because of the risk of choroidal neovascularization [3], the patient has been followed every 3 months during the first year. No CNV occurred at the 3 months examination.

## Discussion and conclusions

As far as we know, we report the first case of macular subretinal bleeding on macular LCs in a highly myopic patient in immediate post partum.

LCs are uncommon findings in the posterior pole of highly myopic eyes, with a prevalence of 4.2 % to 15,7 % [6, 7].

They seem to be associated to higher refractive error, worse visual prognosis, longer axial length, and decreased choroidal thickness [8]. They are described in younger patients, with mean age of 45 years [3]. With time, they are believed to increase in number and width [3].

Morphologically, they appear as fine, irregular, yellow lines, often branching and criss-crossing all over the posterior fundus [9]. LCs correspond to mechanical breaks in the RPE-BM-CC complex and are supposed to be the result of stretching of these tissues secondary to progressive posterior segment elongation [9].

LCs location at the temporal edge of macula is the most frequently described [3]. They must be differentiated from angioid streaks (AS) and myopic stretch lines which are two distinct conditions affecting Bruch membrane [10, 11]. However, these lesions present with distinctive features on multimodal imaging [10–12]. Few studies described the histopathologic features of lacquer cracks [7, 13, 14]. Querques et al. [13] found an association between the position of perforating scleral vessels and lacquer cracks in pathologic myopia. The authors hypothesized that scleral expansion at the site of the perforating scleral vessels could play a role in the formation of lacquer cracks. LCs are often found simultaneously with subretinal hemorrhages [14], as in the present case. It has been suggested that subretinal bleeding may precede the development of a new rupture of Bruch’s membrane and choriocapillaris complex in eyes with pathologic myopia [7, 14]. Subretinal hemorrhages secondary to LCs typically have good prognosis, with complete resorption of the subretinal bleeding in few months [15].

However, a close follow up is required because of the risk of choroidal neovascularization or the occurrence of patchy chorioretinal atrophy [3, 15].

For many years it was thought that increased effort during labor would increase the risks of rhegmatogenous retinal detachment in highly myopic patients [4, 5, 16]. Nonetheless, subsequent studies revealed no evidence of association between vaginal delivery and rhegmatogenous retinal detachment or retinal degeneration [4, 5].

In this case, the macular subretinal bleeding may be explained by a sudden increase of intraocular pressure due to the Valsalva maneuvers in a mechanically weakened area of the globe. Also, we cannot exclude this subretinal bleeding as the natural course of pathologic myopia.

The timelapse of 2 days between the delivery and the ophthalmologic examination may be attributed to the immediate post partum context and delayed access to ophtalmologic emergencies.

Some cases of traumatic lacquer cracks have been reported following photodynamic therapy [17] or laser treatment of choroidal neovascularization [18].

Therefore, we can only speculate that traumatic events like a Valsalva maneuver occurring during labour in this case could explain the pathogenesis of acute subretinal hemorrhage secondary to lacquer cracks.

However, no association between LC and uncomplicated vaginal delivery is described in the medical literature. The macular hemorrhage could be simply the natural evolution of the disease. No causal relationship with delivery can be proven from this sequence of events. The sudden loss of BCVA and therefore a prompt diagnosis may be explained by the foveal location of the LC in our patient.

## Data Availability

All data generated and analyzed during this study are included in this published article and its supplementary information files.

## Abbreviations

AS: Angiod streaks
BM: Bruch’s membrane
BVCA: Best-corrected visual acuity
CC: Choriocapillaris
CNV: Choroidal neovascularization
FA: Fluorescein Angiography
HM: High myopia
ICGA: Indocyanin green angiography
LC: Lacquer cracks
LE: Left eye
MM: Myopic maculopathy
PM: Pathologic myopia
RE: Right eye
RPE: Retinal pigment epithelium
SD-OCT: Spectral domain optical coherence tomography
